# Multiple strategies to improve the yield of chitinase a from *Bacillus licheniformis* in *Pichia pastoris* to obtain plant growth enhancer and GlcNAc

**DOI:** 10.1186/s12934-020-01440-y

**Published:** 2020-09-15

**Authors:** Wen Song, Nuo Zhang, Mo Yang, Yuling Zhou, Nisha He, Guimin Zhang

**Affiliations:** State Key Laboratory of Biocatalysis and Enzyme Engineering, School of Life Sciences, Hubei University, Wuhan, 430062 China

**Keywords:** Chitinase, *Pichia pastoris*, Colloidal chitin, GlcNAc, Plant growth

## Abstract

Chitinase and chitin-oligosaccaride can be used in multiple field, so it is important to develop a high-yield chitinase producing strain. Here, a recombinant *Pichia pastoris* with 4 copies of *ChiA* gene from *Bacillus licheniformis* and co-expression of molecular chaperon *HAC1* was constructed. The amount of recombinant ChiA in the supernatant of high-cell-density fermentation reaches a maximum of 12.7 mg/mL, which is 24-fold higher than that reported in the previous study. The recombinant ChiA can hydrolyze 30% collodidal chitin with 74% conversion ratio, and GlcNAc is the most abundant hydrolysis product, followed by *N*, *N*′-diacetylchitobiose. Combined with BsNagZ, the hydrolysate of ChiA can be further transformed into GlcNAc with 88% conversion ratio. Additionally, the hydrolysate of ChiA can obviously accelerate the germination growth of rice and wheat, increasing the seedling height and root length by at least 1.6 folds within 10 days.

## Introduction

Chitin is the second abundant bioresource that is widely distributed in wastes from shellfish, krill processing, fungal fermentations and green alga. It is constituted of β(1 → 4)-linked *N*-acetyl-d-glucosamine (GlcNAc) units [1]. Chitin can be degraded by chitinases, which takes place in 2 steps, the initial cleavage of the chitin polymer by chitinases into chitin-oligosaccharide and further cleavage to GlcNAc by β-*N*-acetylglucosaminidase [2]. Chitin-oligosaccharide can promote plant growth and induce crops to produce disease resistance factors, effectively resist the infection of plant pathogens [3, 4]. GlcNAc can prevent bone and joint diseases, promote wound healing, and be as antibacterial and anti-inflammatory agent [5, 6]. Therefore, it can be widely used in the industry of medicine, health care, food and cosmetics. At present, the chitin-oligosaccharide and GlcNAc are mainly prepared from chitin through chemical method, which cause severe environmental issues. Thus, using chitinases to hydrolyze chitin, with the mild reaction conditions and less pollution, has become a popular research direction. So, it is important to develop a high-yield chitinase producing strain to lower the cost of chitin hydrolysis into GlcNAc and chitin-oligosaccharides.

*Bacillus licheniformis* is a GRAS organism, has been used extensively in industry for the production of various enzymes and metabolites [7, 8]. Songsiriritthigul et al. firstly expressed the ChiA from *B. licheniformis* in *Escherichia coli* and characterized the recombinant enzyme. They found that ChiA is thermo and pH-stable, hydrolyzing colloidal chitin into GlcNAc and *N*, *N′*-diacetylchitobiose [9]. However, expression of ChiA in *E. coli* is not suitable for industrial applications for its cell disruption, high cost of inducer and protein purification, etc. Then, Menghiu et al. expressed ChiA extracellularly in *P. pastoris*. The recombinant ChiA was glycosylated and the yield in the supernatant reached 0.506 mg/mL in the 1 L flask, which is fivefold than the one in *E. coli* [10]. However, the yield is still far from the requirement of industrial application, multiple strategies are still needed to increase the expression of recombinant ChiA in yeast.

*P. pastoris* has been widely used to produce heterologous protein with its advantages of secretory expression with less background extracellular protein [11]. Recently, multiple strategies have been developed for efficient expression of heterologous protein in *P. pastoris*, such as optimizing the expression vector, increasing the copy number of target gene, co-expressing molecular chaperone, optimizing the fermentation conditions, and so on [12, 13]. In our previous job, the pHBM905M vector used in this study was constructed based on the pHBM905A plasmid [14] with four major modifications, including the replacement of original AOX1 promoter by d1 + 2 × 201 AOX1 promoter, the replacement of original MFα signal peptide by MF4I-SS signal peptide, and the removal of kanamycin resistance gene by the insertion of heterologous gene during the plasmid construction. In addition, pHBM905M can use the Biobrick method to generate a multi-copy expression cassette in vitro [15, 16]. Moreover, there is no exogenous resistance genes in the recombinant *P. pastoris*, which decreased the spread of antibiotics resistance genes to the environment. Molecular chaperones were also used to increase the expression of heterologous proteins in *P. pastoris*. HAC1 can trigger the unfolded protein response (UPR) and increase the secretion of recombinant proteins which were demonstrated in many studies. For example, co-expression of HAC1 increased the expression of Δ9-Tetrahydrocannabinolic acid synthase 4.1-fold [17]. TRM1 is a transcription factor which is important for the transcriptional activation of genes of methanol utilization pathway in *P. pastoris* [18]. As *P. pastoris* is a methylotrophic yeast, which utilize methanol as the only energy and carbon source. The heterologous gene expression is also driven by methanol-inducible promoters, thus, we speculated that TRM1 can promote the utilization efficiency of methanol, resulting in higher expression level of heterologous protein. ERV29, SEC16 and COG5 are involved in the secretory and trafficking pathway, which have been identified to improve the secretion of recombinant proteins in *S. cerevisiae* [19, 20]. Thus, we proposed that co-expression of those chaperones with the target gene seems to be a general strategy for enhancing the expression of recombinant proteins in *P. pastoris*. In addition, *P. pastoris* has strong aerobic growth preferences and can perform high-cell-density fermentation, which is conducive to large-scale industrial production.

In our study, different strategies were used to optimize the expression level of ChiA, containing chromosome insertion of multiple *ChiA* expression cassettes on the basis of the vector pHBM905M, co-expression with molecular chaperones (HAC1, TRM1, EVR29, SEC16, COG5) and high-cell-density fermentation. The recombinant ChiA can act on 30% (w/v) colloidal chitin with high conversion ratio, and the hydrolysis product can be further transformed into GlcNAc by combining with reported β-*N*-acetylglucosaminidase. Moreover, the plant germination-promoting activity of hydrolysate were also investigated.

## Materials and methods

### Plasmids, strains, chemicals and medium

For gene cloning, *E. coli* XL-gold was purchased from Invitrogen. The *P. pastoris* GS115 strain was used for the heterologous expression. The plasmid pHBM905M was used as expression vector to carry *ChiA* gene, which originally constructed in our group [16]. The plasmid pGAPZB from Invitrogen was used to express *HAC1*, *ERV29*, *SEC16*, *COG5* and *TRM1* constitutively. BMGY (buffered glycerol-complex medium), BMMY (buffered methanol-complex medium), MD (minimal dextrose) media were prepared according to the instruction of *Pichia* Expression Kit (Invitrogen, USA). PTM_1_ (Pichia trace metals) solution was prepared as 6.0 g/L CuSO_4_·5H_2_O, 0.08 g/L NaI, 3.0 g/L MnSO_4_·H_2_O, 0.2 g/L Na_2_MoO_4_·2H_2_O, 0.02 g/L H_3_BO_3_, 0.5 g/L CoCl_2_, 20.0 g/L ZnCl2, 65.0 g/L FeSO_4_·7H_2_O, 0.2 g/L biotin, 5 mL/L H_2_SO_4_. Basal salts medium was prepared as 20 g/L NH_4_H_2_PO_4_, 0.9 g/L CaSO_4_, 12 g/L K_2_SO_4_, 10 g/L MgSO_4_·7H_2_O, 4 g/L KH_2_PO_4_, 0.6 g/L KOH, 40 mL/L Glycerol. The *B. licheniformis* WX-02 was provided by Prof. Shouwen Chen from Hubei University. Based on the sequence in GenBank (Accession Number CP012110.1, 311870-313657 bp), the DNA primers were synthesized by Sangon Biotech Co., Ltd. (Shanghai, China). A Bradford assay kit for protein quantification in the fermentation supernatant was purchased from Beyotime Biotechnology (Shanghai, China). GlcNAc and *N*, *N′*-diacetylchitobiose were purchased from BZ Oligo Biotech Co., LTD (Qingdao, China) as controls. Restriction enzymes, DNA polymerase, DNA Ligation Kit, and other related reagents were bought from Takara (Dalian, China).

### Construction of the multi-copy expression cassette plasmids

The primers used in this study are listed in Table 1. The *ChiA* gene from *B. licheniformis* was amplified by PCR with primers ChiA-F and ChiA-R, then cloned into the expression vector pHBM905M digested by *Cpo*I and *Not*I (Additional file 1: Fig. S1). The recombinant plasmid included single copy of *ChiA* was confirmed by sequencing and named as pHBM905M-*ChiA*-1C (Additional file 1: Fig.S2A). Based on pHBM905M-*ChiA*-1C, other multiple copies of *ChiA* gene were constructed through the biobrick method [21]. The whole expression cassette was cleaved from the plasmid pHBM905M-*ChiA*-1copy using *Xba*I/*Bam*HI, then cloned into the pHBM905M-*ChiA*-1copy with *Spe*I/*Bam*HI digestion to generate pHBM905M-*ChiA*-2copy plasmid, which contained 2 copies of expression cassette. The *Xba*I and *Spe*I are isocaudamer, so the ligation would destroy the original *Spe*I site in the plasmid pHBM905M-*ChiA*-2copy (Additional file 1: Fig. S2A). The plasmids containing 3, 4 and 6 copies of the *ChiA* expression cassettes were constructed by repeating this procedure, then confirmed by plasmid size and *Sal*I digestion (Additional file 1: Fig.S2B), and then transformed into *P. pastoris* GS115.

**Table 1:** The primers used in the study

Primers name	Sequence
ChiA-F	GTCAGATTCCGGAAAAAACTATAAAATCATCGGC
ChiA-R	GGCCATTATTCGCAGCCTCCGATCAGCC
HAC1-F	GAATTCCAATCATTTTTTTTTTTTGTCTGTGTATTCTTCTTA
HAC1-R	CTCGAGATGAAGCCTGCATCTCTCAGG
ERV29-F	GAATTCATGTCTTATCGCCCTCAGTTTCAACA
ERV29-R	CTCGAGTCAATAGATCTTTTTCTTTTCATCAAAACTCAA
SEC16-F	GAATTCATGGTTACAATTGGAAACGCACAT
SEC16-R	CTCGAGTTATTGATCGACTTCGCCAGC
COG5-F	GAATTCATGTCTTTGGAGGACTTTGACGG
COG5-R	CTCGAGTTAGCCATTCAATACCCTAATAGCATTAATCATC
TRM1-F	GAATTCCTATCGCACCTCACTCATAGAAAATC
TRM1-R	CTCGAGGGGGGGGCATTCTAGTATGTACAAATAG

### Expression of ChiA in *P. pastoris* through shake flask fermentation

The five recombinant plasmids (pHBM905M-*ChiA*-1copy, pHBM905M-*ChiA*-2copy, pHBM905M-*ChiA*-3copy, pHBM905M-*ChiA*-4copy and pHBM905M-*ChiA*-6copy) were linearized by *Sal*I and electroporated into *P. pastoris* GS115. All transformants were selected on MD plates containing 0.4 mg/L biotin without histidine, and then identified by PCR to amplify the *ChiA*. The recombinant *P. pastoris* strains, A1C, A2C, A3C, A4C and A6C, were cultured respectively in 50 mL BMGY medium until the OD_600_ reached 20–25. Then the yeast cells were transferred into 25 mL BMMY medium, and incubated at 28 °C. We added 125 μL methanol to induce target protein expression every 12 h, with a total induction time of 7 days. Every 24 h, the cell culture were collected for measurement of enzyme activity and analyzed on a 12% SDS-PAGE.

### Co-expression of ChiA with molecular chaperones

The *HAC1*, *ERV29*, *SEC16*, *COG5*, and *TRM1* genes from *P. pastoris* GS115 were amplified by PCR (Primers in Table 1), and cloned into the pGAPZB vector respectively (Additional file 1: Fig. S3). Then, these constitutive expression vectors were linearized with *Avr*II and transformed individually into *P. pastoris* A4C, and the transformants were selected on YPD plates with 100 µg/mL zeocin. The right recombinant strains were verified using colony PCR, respectively. The *P. pastoris* A4C, co-expression with HAC1, ERV29, SEC16, COG5, and TRM1were cultured respectively in 50 mL BMGY medium until the OD_600_ reached 20–25. Then the yeast cells were transferred into 25 mL BMMY medium, and incubated at 28 °C. We added 125 μL methanol to induce target protein expression every 12 h, with a total induction time of 7 days. Every 24 h, the cell culture were collected for measurement of enzyme activity and analyzed on a 12% SDS-PAGE.

### Analysis of chitinase activity on colloidal chitin

DNS method was used to measure the activity of ChiA towards colloidal chitin [22]. The colloidal chitin was prepared as previously described [23]. The fermentation supernatant was collected by centrifuging at 10,000*g* for 5 min at 4 °C. The mixture with 500 μL 2% (w/v) colloidal chitin and 50 μL fermentation supernatant was incubated at 50 °C for 10 min, then 500 μL DNS was added and the mixture was incubated at 100 °C for 15 min. The mixture was centrifuged at 10,000*g* for 1 min at room temperature to precipitate the non-hydrolyzed chitin, and the supernatant was transferred to a cuvette and the absorbance at 540 nm was measured to quantify the concentration of reducing sugars. One unit of ChiA activity is defined as the quantity of enzyme needed to release 1 μM GlcNAc per minute.

### High-cell-density fermentation of A4C co-expressing with *HAC1*

In order to improve the ChiA expression level continually, we used 5 L high-cell-density fermentation according to the *Pichia* fermentation process guidelines (Invitrogen), which included three stages [24]. Firstly, a fresh colony was inoculated into 200 mL YPD medium, incubated at 28 °C, and 200 rpm for 48 h, then transferred into 5 L fermentor with 2 L of the basal salt medium with 8 mL/L PTM_1_ solution to keep the initial OD_600_ about 0.5. During the cell growth stage, the temperature was set at 28 °C, and the pH was maintained at 6.0 by adding ammonia hydroxide. The cells were allowed to grow until the glycerol was exhausted, as indicated by an increase of DO (Dissolved oxygen) level. The glycerol was exhausted after 29 h, DO increased to 60% rapidly. Then the second stage was initiated by feeding 50% (w/v) glycerol containing 12 mL/L PTM_1_ solution, and the feeding rate was varied to maintain the DO level at 20–30%. When the OD_600_ of cells reached 300, the glycerol feeding was stopped, DO rose again. About 30 min later, the third stage of the fermentation began with the feeding of pure methanol containing 12 mL/L PTM_1_ solution into the fermenter to induce the expression of the target gene, and the feeding rate was varied to maintain the DO level at 20–30%. The fermentation temperature was adjusted to 22 °C [16]. Samples were collected every 12 h from cell culture to determine the OD_600_ and enzyme activity.

### Recombinant ChiA hydrolyzes colloidal chitin at different concentrations

The reaction mixtures, including 200 µL ChiA (18 U/mL) mixed with 500 µL 10%, 15%, 25%, 30%, 35% and 40% colloidal chitin respectively were incubated at 50 °C for 12 h. The reaction products were determined by high performance liquid chromatography (HPLC), using Nexera UHPLC/HPLC System with a Zorbax Carbohydrate Analysis Column (4.6 × 250 mm, 5 µm) (Agilent, America). The mobile phase consisted of acetonitrile and water (70:30, v/v). The flow rate was 0.5 mL/min. Products were detected at 190 nm. The injection volume was 20 µL. The DNS method was used to determine the reducing sugar concentration, and the conversion ratio was calculated by comparing the quantity of actual reducing sugars over the actual colloidal chitin dry weight. The optimum colloidal chitin concentration is determined by comparison of conversion ratio and the amount of reaction products by HPLC.

### ChiA hydrolyzing colloidal chitin combined with BsNagZ to obtain GlcNAc

The reaction mixture including 300 µL 1 mM *N*, *N*’-diacetylchitobiose and 50 µL ChiA (18 U/mL) was incubated at 50 °C for 20 min. The reaction products were also analyzed by HPLC. The hydrolysis ratio of *N*, *N*′-diacetylchitobiose was calculated according to the standard curve drafted by the peak areas of standard and each experiment was repeated three times to minimize the experimental deviation. The hydrolysis rate of *N*, *N*′-diacetylchitobiose (%) = released GlcNAc (mg) × 100%/Original *N*, *N*′-diacetylchitobiose (mg).

BsNagZ is β-*N*-acetylglucosaminidase from *B. subtilis*, which has been identified to cut the colloidal chitin from the terminal to obtain GlcNAc with low efficiency, combining commercial chitinase can improve the yield of GlcNAc efficiently in our previous work [25]. Here, a mixture of 600 µL of 30% (w/v) colloidal chitin and 200 µL ChiA (18 U/mL) was kept at 50 °C for 12 h, and 200 µL BsNagZ (5 U/mL) was added and the mixture further incubated at 60 °C for another 20 min to obtain GlcNAc. The reaction products were then analyzed by HPLC, and GlcNAc was quantified using the standard curve. The conversion ratio (%) = released GlcNAc (mg) × 100%/the colloidal chitin dry weight (mg).

### Influence of chitin-oligosaccharides on seed germination and seedling growth of rice and wheat

In order to explore the optimal concentration of chitin-oligosaccharides to promote the germination of rice and wheat. Fifty grains of rice and wheat seeds of uniform size were selected and placed in a Petri dish, respectively. The rice seeds were soaked with 50 μg/mL, 150 μg/mL, 300 μg/mL and 500 μg/mL ChiA 12 h-hydrolyzed products on colloidal chitin respectively, likewise, 1 μg/mL, 10 μg/mL, 25 μg/mL and 100 μg/mL hydrolysate for the wheat seeds, and the distilled water as the control. The samples were hydrated every day with a sprayer, and germinated at room temperature for 7–10 days to observe the germination and growth of rice and wheat seeds.

Then, another fifty grains of rice and wheat seeds of uniform size were selected and placed in a Petri dish. Since colloidal chitin is dissolved in 0.1 M pH 6.0 phosphate buffer, we also use phosphate buffer as a control. The seeds were then soaked at room temperature with water, phosphate buffer (pH 6.0), ChiA 12 h-hydrolyzed products on colloidal chitin and ChiA/BsNagZ hydrolyzed products on colloidal chitin (300 μg/mL for rice seeds, 10 μg/mL for wheat seeds), respectively. The samples were hydrated daily with a sprayer, and germinated at room temperature for 7–10 days to measure the root and bud length. The average value and variance of each group were calculated and Student’s *t* test was used to analyze differences between experiment group and control group.

## Results

### Induced expression of *ChiA* in *P. pastoris*

**Fig. 1:**
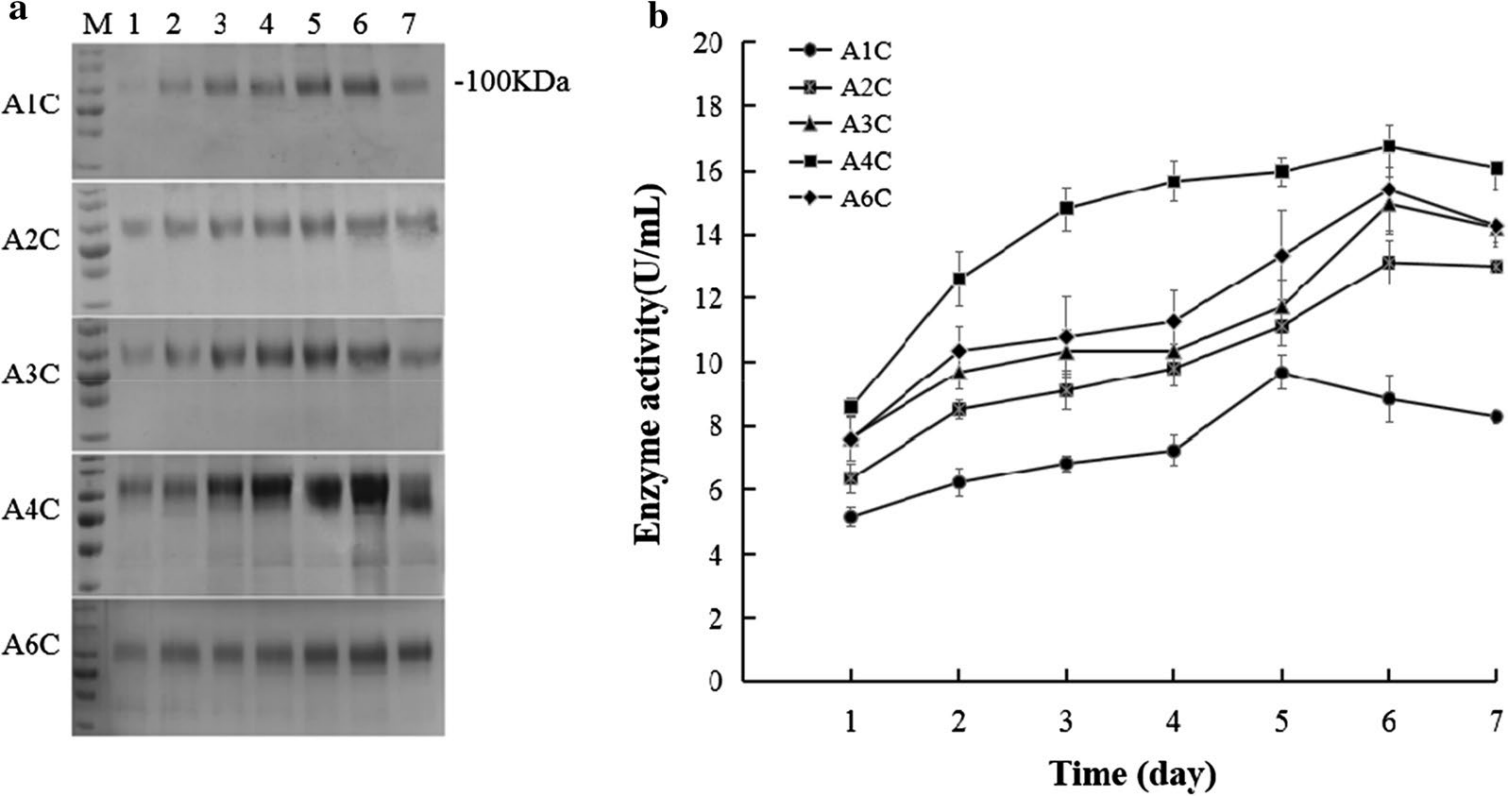
**a** SDS-PAGE analysis of fermentation supernatant containing recombinant ChiA from *P. pastoris*. M: Protein molecular marker. lanes 1–7: Fermentation supernatant (8 μL) after 1–7 days induction, respectively. **b** Chitinase activities of fermentation supernatant after 1–7 days induction

The molecular weight of ChiA is 76.20 kDa predicted by the ExPASy Bioinformatics Resource program, and the ChiA has 7 potential N-glycosylation sites. The recombinant ChiA was previously identified as an glycosylated enzyme in *P. pastoris* through deglycosylation treatment [10]. Here, the recombinant ChiA migrated as an about 100 kDa band through SDS-PAGE analysis (Fig. 1a), same as the previous report [10]. As the copy of gene increases, the secretory expression of ChiA also improved from the strain A1C to A4C, while the strain A6C secreted the less ChiA (Fig. 1a). Activity analysis showed that *P. pastoris* A4C gave the highest enzyme activity and expression level on the 6th day of induction, reaching 16.74 U/mL (1.13 mg/mL), higher than the strain with 1, 2, 3 and 6 copies of ChiA on the 6th day with 8.84 U/mL (0.53 mg/mL), 13.13 U/mL (0.84 mg/mL), 14.20 U/mL (1.05 mg/mL) and 15.41 U/mL (1.07 mg/mL) respectively (Fig. 1b).

### Co-expression of molecular chaperones to improve the yield of ChiA

**Fig. 2:**
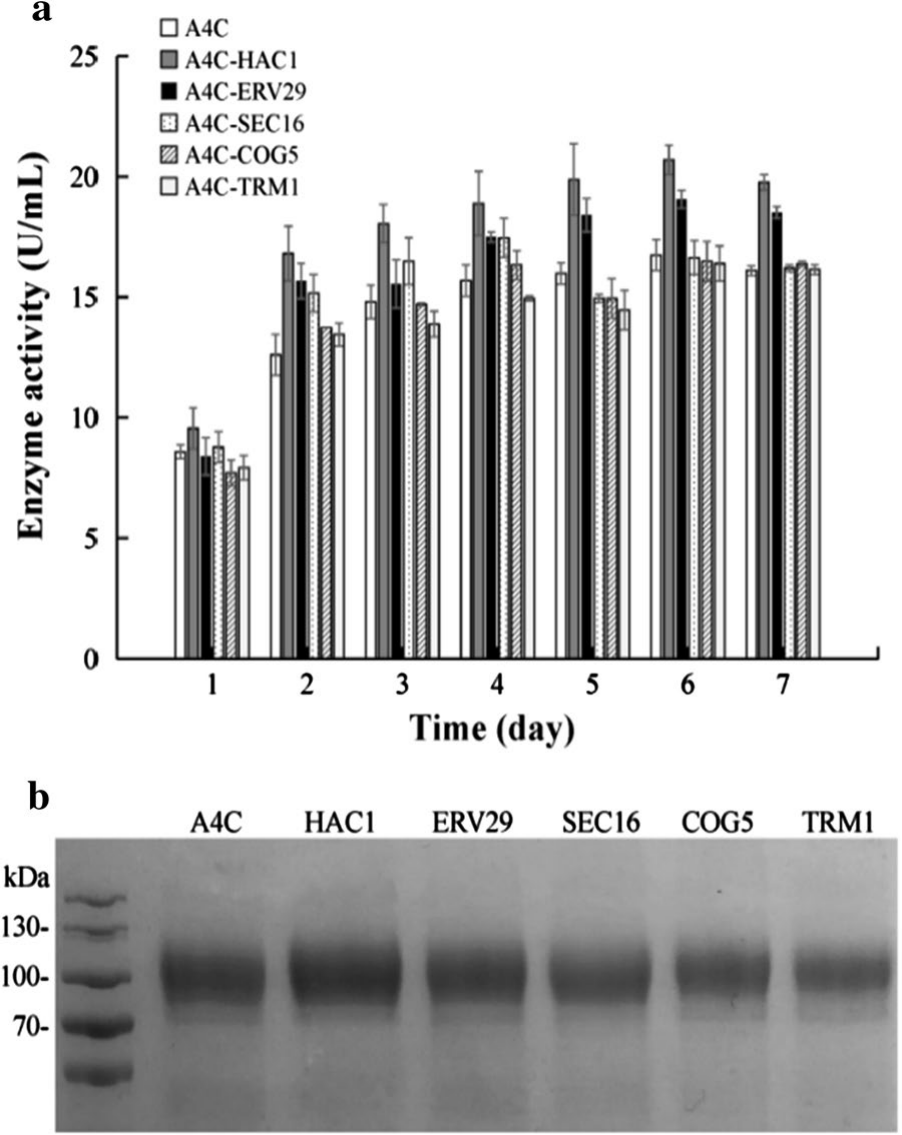
Chitinase activities and SDS-PAGE analysis of fermentation supernatant of A4C with different chaperones. **a** Chitinase activities in the fermentation supernatant. **b** SDS-PAGE analysis of fermentation supernatant (8 μL). lane 1: A4C, lanes 2–6: A4C co-expression of *HAC1*, *SEC16*, *ERV29*, *COG5* and *TRM1*, respectively

The *Avr*II-linearized vectors pGAPZB-*HAC1*/*ERV29*/*SEC16*/*COG5*/*TRM1* was transformed into *P. pastoris* A4C, respectively. Co-expression of *HAC1* and *ERV29* separately increased the ChiA yield in the fermentation supernatant of flask from 16.74 U/mL (1.13 mg/mL) to 20.69 U/mL (1.40 mg/mL) and 19.00 U/mL (1.26 mg/mL), respectively (Fig. 2a). However, co-expression of *SEC16*, *COG5* or *TRM1* didn’t give obvious promotion for ChiA expression (Fig. 2). We also tried to express *HAC1* and *ERV29* simultaneously in A4C strain, yet the activity did not further increase compared with co-expression of single chaperone. Thus, co-expression of *HAC1* can give the highest activity of 20.7 U/mL.

### Expression of ChiA using high-cell-density fermentation

**Fig. 3:**
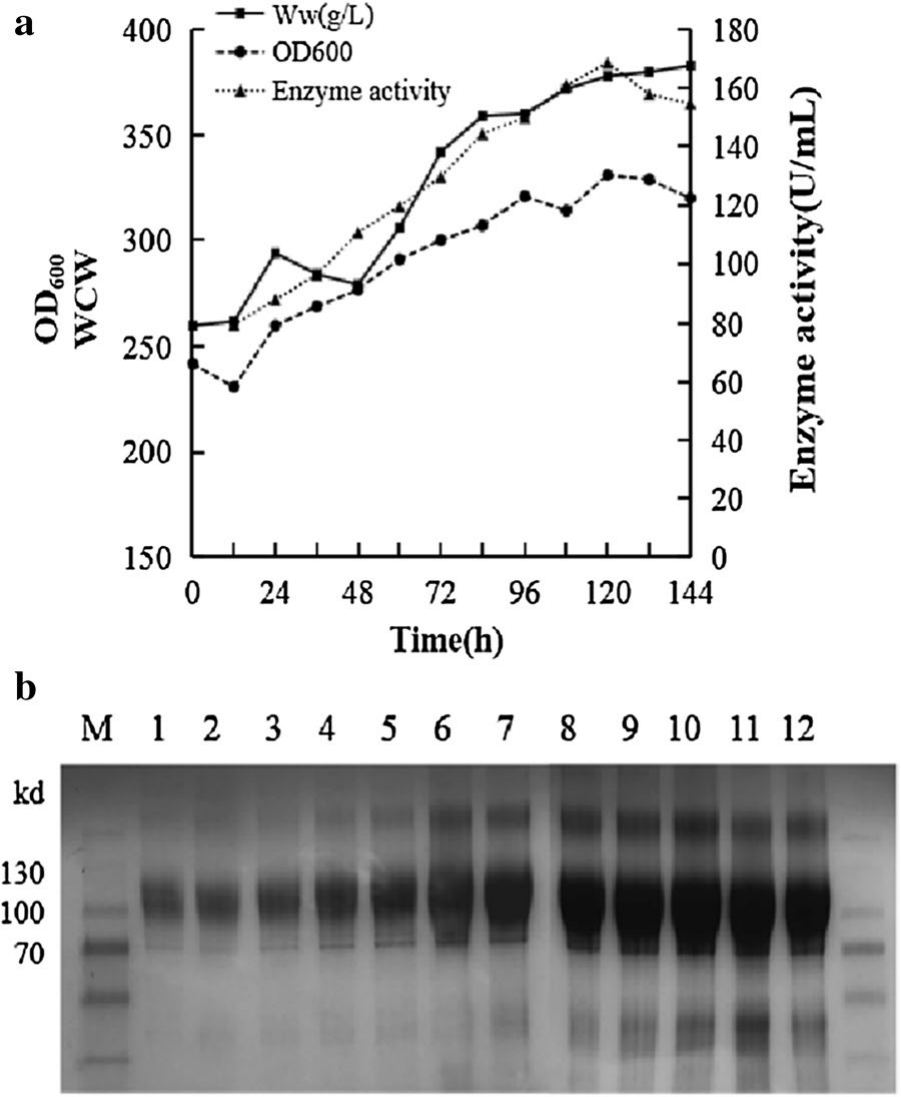
The high-cell-density fermentation of the recombinant *P. pastoris* A4C co-expressing *HAC1* in a 5 L fermenter. **a** The growth curve (OD_600_), WCW and chitinase activity of fermentation supernatant with different induction time. **b** SDS-PAGE analysis of fermentation supernatant (8 μL) with different induction time. lane M, Prestained protein marker; lanes 1–12, The fermentation supernatants after being induced for 0, 12, 24, 36, 48, 60, 72, 84, 96, 108, 120 and 132 h, respectively

In order to further improve the expression level of ChiA, high-cell-density fermentation of A4C-*HAC1* was performed in a 5 L fermenter. The samples were collected every 12 h, and the whole methanol induction time was 144 h. The maximum enzyme activity in the supernatant reached 168.78 U/mL after 120 h induction, eightfold higher than in shake flask, and the amount of secreted ChiA kept increasing to reach a maximum of 12.70 mg/mL (Fig. 3a), which was ninefold higher than the shake flask fermentation (1.40 mg/mL). When the enzyme activity reached the highest, the final cell density of OD_600_ and wet weight reached 331 and 378  g/L, respectively.

### Recombinant ChiA hydrolyzing colloidal chitin at different concentrations

**Fig. 4:**
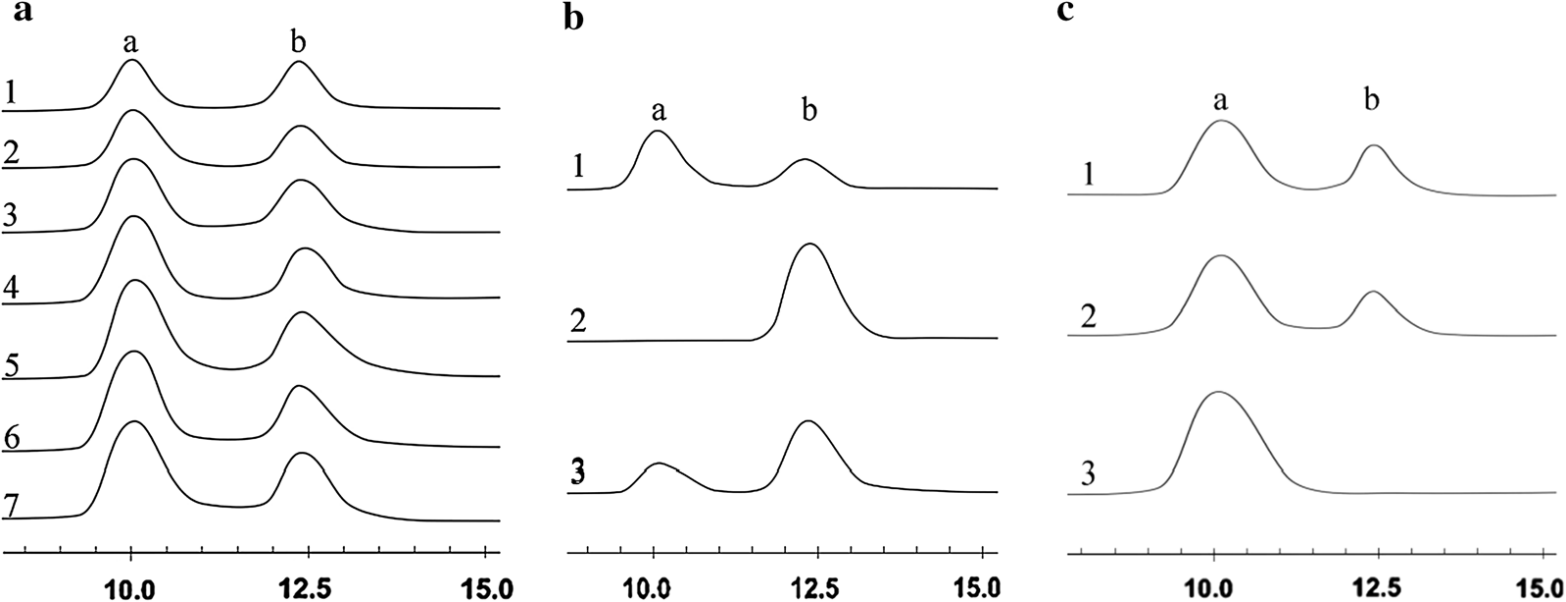
Analysis of hydrolysis products by HPLC. **a** The hydrolysis products of colloidal chitin with different concentration by recombinant ChiA for 12 h. 1: Standard of GlcNAc (peak a) and *N*, *N′*-diacetylchitobiose (peak b). 2–7: 10%, 15%, 25%, 30%, 35%, 40% colloidal chitin as substrate, respectively. **b** The hydrolysis products of *N*, *N*′-diacetylchitobiose by recombinant ChiA. 1: Standard of GlcNAc (peak a) and *N*, *N*′-diacetylchitobiose (peak b). 2, 3: The hydrolysis product of *N*, *N*′-diacetylchitobiose by recombinant ChiA at 0, 20 min, respectively. **C** The hydrolysis products of colloidal chitin by recombinant ChiA/BsNagZ. 1: Standard of GlcNAc (peak a) and *N*, *N*′-diacetylchitobiose (peak b). 2, 3: The hydrolysis product of colloidal chitin by recombinant ChiA for 12 h and then with recombinant BsNagZ for 0 (2), 20 min (3)

Menghiu et al. determined the hydrolysis products of recombinant ChiA from *P. pastoris* by TLC using 2% colloidal chitin as substrate, which showed the reaction products were mainly *N*, *N*′-diacetylchitobiose, followed by GlcNAc, and considered that ChiA is an endochitinase [10]. As we known, high substrate concentration can give more products in a limited reaction volume, however, high concentration products may inhibit the enzymatic reaction. Thus, in order to explore the optimal concentration of colloidal chitin hydrolyzed by recombinant ChiA, we tried to use excessive ChiA hydrolyze different concentration colloidal chitin. The results showed that the conversion ratio of 10%, 15%, 25%, 30%, 35% and 40% (w/v) colloidal chitin can reach 89%, 80%, 78%, 74%, 69% and 62%, respectively. When hydrolyzed 30% (w/v) colloidal chitin, the yield of products looks more higher than the others by HPLC analysis (Fig. 4a). When the substrate concentration was improved to 40%, the yield didn’t increase. Thus, 30% (w/v) colloidal chitin was selected as the suitable substrate concentration. We also found that more GlcNAc are obtained in the hydrolysate, and *N*, *N*′-diacetylchitobiose gradually decreased, which is different from the previous report that *N*, *N*′-diacetylchitobiose as the most abundant hydrolysis product by the recombinant ChiA from *P. pastoris* [10], and consistent with the report that colloidal chitin was hydrolyzed by the recombinant ChiA from *E. coli* up to 3 days at 50 °C, GlcNAc gradually increased [9]. These results implied that *N*, *N*’-diacetylchitobiose can be hydrolyzed by ChiA to GlcNAc, while no previous reports investigated whether this enzyme can hydrolyze *N*, *N*′-diacetylchitobiose. Thus, the *N*, *N*′-diacetylchitobiose was used as a substrate to confirm whether the ChiA have the hydrolytic ability on it. We found *N*, *N*′-diacetylchitobiose can be hydrolyzed by ChiA into GlcNAc weakly, the conversion ratio is 34% even the excessive ChiA was used (Fig. 4b).

### ChiA/BsNagZ hydrolyzing colloidal chitin to obtain GlcNAc

β-*N*-acetylglucosaminidase BsNagZ has been identified to cut the colloidal chitin from the terminal to obtain GlcNAc at low efficiency [25]. Here, a mixture of 600 µL of 30% (w/v) colloidal chitin and 200 µL ChiA (18 U/mL) was incubated at 50 °C for 12 h, and then 200 µL BsNagZ (5 U/mL) was added and the mixture was kept at 60 °C for another 20 min. The results showed that GlcNAc is the only product, it can be produced efficiently when BsNagZ was used as a supplement (Fig. 4c), and the conversion ratio can reach 88%.

### The effects of different hydrolysis products on the germination of rice and wheat seeds

**Fig. 5:**
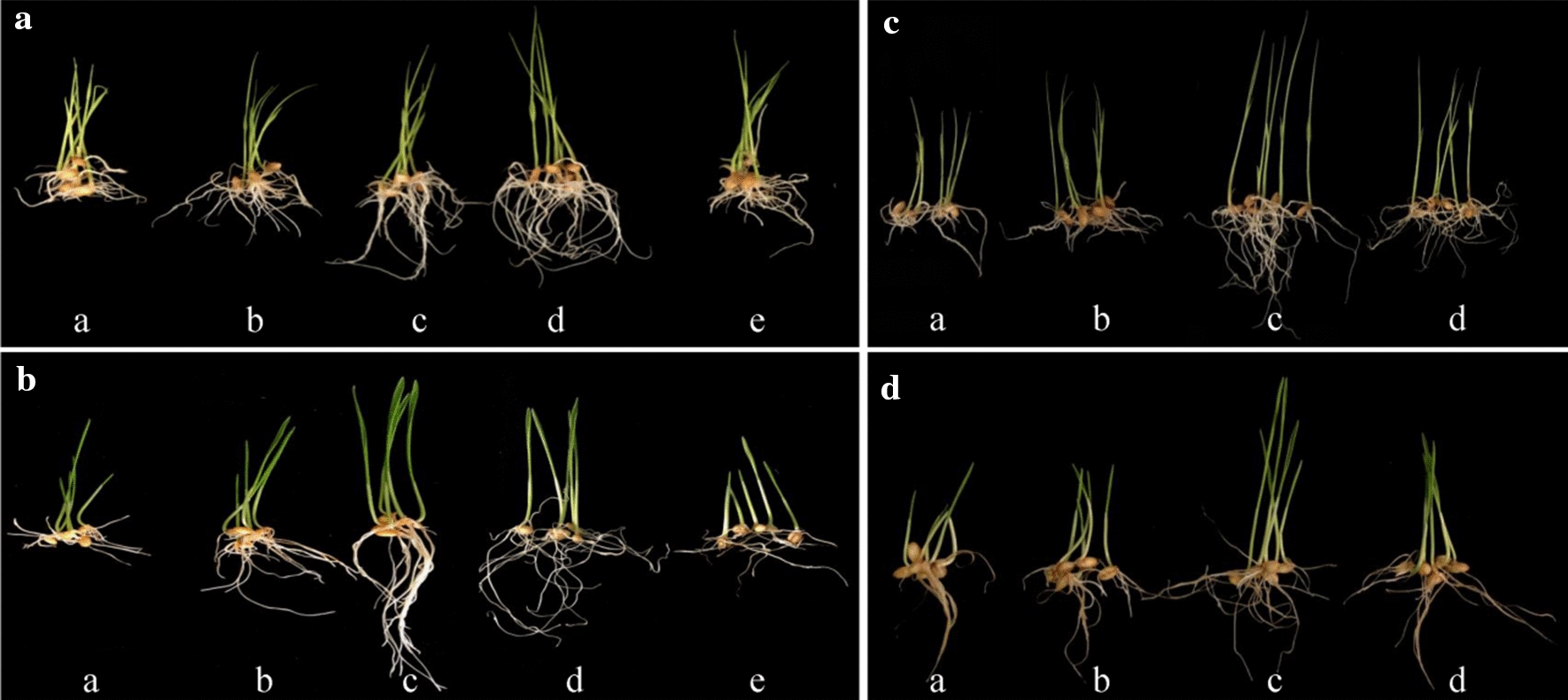
Effects of the hydrolysis products on the germination growth of plants. **a** Different concentration hydrolysis products by ChiA on rice seeds germination. a–e water, 50 μg/mL, 150 μg/mL, 300 μg/mL and 500 μg/mL. **b** Different concentration hydrolysis products by ChiA on wheat seeds germination. a-e: water, 1 μg/mL, 10 μg/mL, 25 μg/mL and 100 μg/mL. **c** Effects of different hydrolysis products on rice seed germination. (a) water, (b) pH6 phosphate buffer, (c) 300 μg/mL ChiA hydrolyzed colloidal chitin product, (d) 300 μg/mL ChiA/BsNagZ hydrolyzed colloidal chitin product (monosaccharide). **d** Effects of different hydrolysis products on wheat seed germination. (a) water, (b) pH6 phosphate buffer, (c) 10 μg/mL ChiA hydrolyzed colloidal chitin product, (d) 10 μg/mL ChiA/BsNagZ hydrolyzed colloidal chitin product (monosaccharide)

Different chitin-oligosaccharides have different optimal concentrations for different crops [26], in our experimental condition, we found ChiA 12 h-hydrolyzed products on colloidal chitin (a mixture of *N*, *N*’-diacetylchitobiose and GlcNAc) with a concentration of 300 μg/mL (Table 2, Fig. 5a) had the best effect for rice germination, and 10 μg/mL for wheat (Table 2, Fig. 5b). When the concentration of chitin-oligosaccharides increased further, the effects of growth promotion of rice and wheat became worse.

**Table 2:** The effects of hydrolyzate by ChiA with different concentration on the germination of rice and wheats seeds

Plant	Concentration (μg/ml)	Seeding length (cm)	Increasing percent (%)	Root length (cm)	Increasing percent (%)
Rice	0	3.5 ± 0.5	–	3.2 ± 0.9	–
	50	4.9 ± 0.9	40	4.1 ± 0.3	28
	150	6.0 ± 0.7**	71	6.1 ± 1.6	190
	300	6.5 ± 0.6**	86	6.3 ± 0.7*	197
	500	5.8 ± 0.5**	66	3.8 ± 0.9	18
Wheat	0	3.8 ± 0.9	–	3.2 ± 0.6	–
	1	5 ± 1.3	32	4.8 ± 1.1	50
	10	6.8 ± 0.3**	79	7 ± 0.7**	119
	25	5.5 ± 0.3	45	5.8 ± 0.3**	81
	100	3.3 ± 1.2	− 13	3.6 ± 0.9	12

*P < 0.05(significant difference), **P < 0.01(Extremely significant difference)

**Fig. 6:**
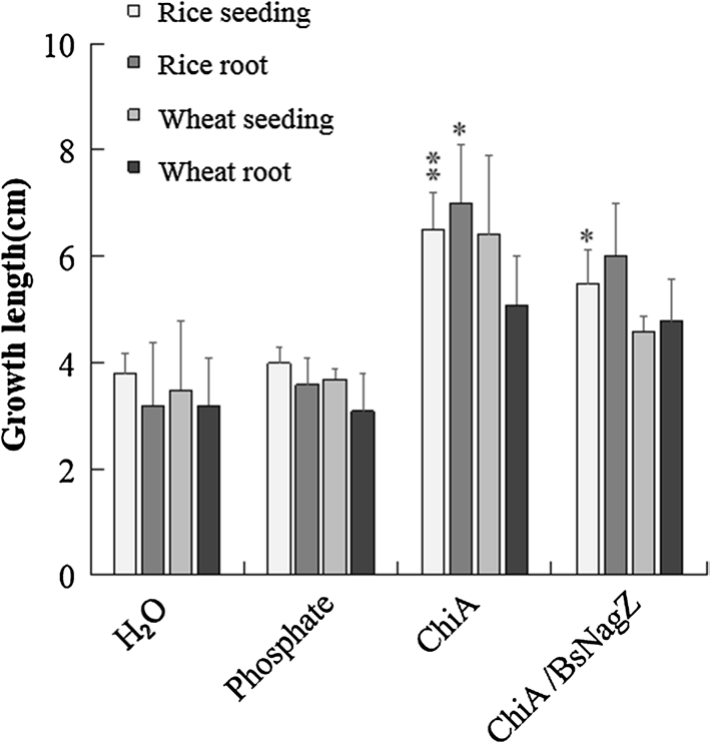
The effects of different hydrolyzate on the germination of rice and wheats seeds. Bars indicated mean ± SD (n = 3 biological replicates); T-tests were noted as *P < 0.05 or **P <  0.01. ChiA and ChiA/BsNagZ means hydrolysis products by ChiA or ChiA/BsNagZ respectively

ChiA/BsNagZ hydrolyzed products on colloidal chitin (monosaccharide) were also diluted to the suitable concentration to evaluate the effects on seeds germination. The phosphate buffer was used as a control. The results showed that the hydrolysis product of ChiA can increase the seedling height and root length up to 1.7-fold and 2.2-fold on rice (Fig. 5c), and up to 1.8-fold and 1.6-fold on wheat (Fig. 5d), respectively (Additional file 1: Table S1). The monosaccharide obtained from ChiA/BsNagZ combined hydrolysis can increase the seedling height and root length of rice up to 1.5-fold and 1.9-fold (Fig. 5c), and up to 1.3-fold and 1.5-fold for wheat (Fig. 5d), respectively (Fig. 6, Additional file 1: Table S1).

## Discussion

In previous study, the chitinase A from *B. licheniformis* has been expressed in the *P. pastoris* firstly. The maximum yield of ChiA in the supernatant of flask is only 0.506 mg/mL [10]. In our study, the multi-copy strategy was used to improve the expression obviously. The strain with 4 copies of *ChiA* gene produced the highest enzyme activity of 16.74 U/mL (1.13 mg/mL) in the supernatant of flask, compared with the 1, 2, 3 and 6 copies of *ChiA* gene. From our results, the copy number of the gene is not proportional to the amount of expressed protein (Additional file 1: Fig. S4). Similar results in different protein cases have been reported before. The recombinant lactone hydrolase ZHD from *Gliocladium roseum* has been expressed in *P. pastoris*. The recombinant *P. pastoris* X3C contained 3 copies of *ZHD* present higher expression than the one with the 4 copies [12]. When 1–4 copies of *ophcM*-expressing cassettes were transformed into *P. pastoris*, 2-copy tandem *ophcM*-expressing cassettes produced the maximum yield [13]. The reason may be that the high gene copy number will intense the cellular stress and reduce the extracellular secretion of the protein [27]. The copy number of gene is not directly proportional to the expression level of protein. Excessive gene copy number may decrease the protein expression level. There are three main speculations in the published papers, the first one is high gene copy number may lead to metabolic burden on the level of central carbon metabolism through metabolic flux analysis, thus limitation in the carbon and energy supply lower the protein level [28]. The second is transcriptome analysis showed that increased dosage of AOX1 promoter-regulated expression cassettes leads to transcription attenuation of the methanol utilization genes, and subsequent experiment also found reduced methanol consumption rates, which may decrease the translation of target protein [29]. The third is mass expressed heterologous protein may cause stress on the secretory pathway, leading to the upregulation of the unfolded protein response (UPR) and resulting in degradation of the protein, which will cause secretion saturation [27]. Since HAC1 can trigger the unfolded protein response (UPR), co-expression of HAC1 can improve the secretion of target protein in our and many other studies [27, 30], which also support this speculation. Actually, there is no definite evidence to explain how the gene copy number affect the corresponding expression level, it is a long way to go.

Molecular chaperones were reported to be an effective method to improve the expression of heterologous protein since they can facilitate unfolded or partially folded protein to be correct folding, finally improve the properties of protein. Here five molecular chaperones *HAC1*, *EVR29*, *SEC16*, *COG5* and *TRM1* have been co-expressed separately with 4 copies of *ChiA* in *P. pastoris*. It is the first time to try co-expression of *TRM1, EVR29* and *COG5* with heterologous protein in *P. pastoris*. Co-expressing *ChiA* with *SEC16, COG5* and *TRM1* separately did not improve the ChiA expression significantly. Here co-expression of *HAC1* and *ERV29* separately increased the ChiA yield from 16.74 to 20.69 U/mL and 19.00 U/mL in the fermentation supernatant of flask. Huang et al. reported that overexpressed *ERV29* or *COG5* individually could improved the secretion level of amylase in *S. cerevisiae.* It is likely that the improved activity of amylase in the *ERV29* overexpressed strain was related to enhancement of the ERV29p-dependent transport. When the *COG5* were overexpressed, the retrograde vesicles trafficking strengthened, which was beneficial for amylase production [31]. However, the co-expression of *SEC16* and *COG5* promotes the secretion of heterologous proteins for the first two days in this study, then has little effect afterwards. We speculate that the reason may be caused by the shorter period of induced protein expression in *S. cerevisiae*, while *P. pastoris* requires longer induction time. Strangely, overexpression of *HAC1* and *ERV29* simultaneously did not further increase expression compared with that of single chaperone expression, which may due to its redundant function.

Meanwhile, our study used high-cell-density fermentation to express the recombinant ChiA for the first time. In our work, the maximum expression level in the supernatant of high-cell-density fermentation reached 12.70 mg/mL after 120 h methanol induction and the activity toward colloidal chitin was 168.78 U/mL, which was tenfold higher than that of shake flask fermentation. In the previous report, the expression of ChiA in *P. pastoris* is 0.506 mg/mL in the 1 L flask supernatant and the activity toward colloidal chitin was 1.70 U/mL. When expressed in *P. pastoris*, the activity was measured using 84 mg/mL (8.4%) colloidal chitin, incubated at 37 °C for 2 h [10]. In our study, we used 2% colloidal chitin at 50 °C for 10 min. In our knowledge, chitin purchased from different companies have a great impact on the measurement of enzyme activity, so we cannot compare the enzyme activity accurately. However, the protein concentration of chitinase (12.70 mg/mL) was about 24-fold higher than the previous report (0.506 mg/mL) [10]. Thus, combining with the multi-gene copy tandem and molecular chaperone co-expression, the high-cell-density fermentation demonstrated to be an efficient strategy to increase the yield of recombinant chitinase in *P. pastoris*.

Usually, high concentration of substrate would inhibit the enzyme activity, while in industry application, high concentration of substrate can reduce the reaction volume to lower the production cost. In our experimental conditions, the optimal colloidal chitin substrate concentration was 30% (w/v), which was higher than the 84 mg/mL (8.4%) reported before [10], and the conversion ratio was better. When the substrate concentration was more than 30%, the conversion rate no longer significantly increased. To our knowledge, this is the first report that a 74% conversion ratio was achieved even with a high concentration of colloidal chitin at 30%.

Some chitinanses can be potentially applied in the enzymatic preparation of GlcNAc. Sashiwa et al. reported that GlcNAc can be produced from α-chitins with 64–77% yields up to 10 days using *A. hydrophila* H-2330 crude enzyme extracts [32]. Cardozo et al. used the enzyme extracts from marine-derived *Aeromonas* to produce GlcNAc from colloidal α-chitin, the highest GlcNAc yield was 93% after 24 h hydrolysis [33]. All these researches used the crude enzyme to obtain GlcNAc. Shun Jiang et al. reported that commercial chitinase CtnSg can hydrolyze 3% colloidal chitin into *N*, *N*’-diacetylchitobiose, and then hydrolyze into GlcNAc by a heterologous expressed β-*N*-acetylglucosaminidase BsNagZ [25]. In our case, when combined with BsNagZ, ChiA could hydrolyze 30% colloidal chitin to the unique product GlcNAc. The activity of ChiA is much higher than the commercial chitinase CtnSg, the cost will decrease significantly for the industrial application to produce GlcNAc. Thus, the enzyme-coupled system of ChiA would provide the new choice to get more GlcNAc for industrial demand.

It was reported that oligosaccharides, especially chitin-oligosaccharides, can promote the growth of some plant species [34, 35]. In our seed germination experiment, we found that the hydrolysis product of ChiA can increase 71% seedling height and 119% root length of rice, increase 83% seedling height and 59% root length of wheat respectively. The monosaccharide obtained from ChiA/BsNagZ hydrolyze colloid chitin can increase 45% seedling height and 87% root length of rice, increase 31% seedling height and 50% root length of wheat respectively. Yunhong et al. showed 0.05% chitin oligosaccharides (Degree of polymerization 2–8) could promote the formation of shoot-borne roots of wheat, but there is no obvious growth-promoting effect on wheat seedlings [36]. Jianxin et al. used the chitinase from *Arthrobactor* sp. to hydrolyze 1% colloidal chitin, the hydrolysate (Degree of polymerization 2–6) with low concentration can increase 39% seedling height of rice while the root growth was slightly inhibited. Otherwise, the growth of rice roots and seedlings were inhibited by the hydrolysate with high concentration [37]. Hongjuan et al. reported 1 μg/mL chitosan (MW 2kD) can significantly increase the growth of wheat root and seedling to 19.2% and 23.1%, while 10 μg/mL chitosan inhibit their growth [38]. In all, different composition and content of oligosaccharides have different effects on plant growth [26]. The hydrolysis products used in our experiment only contains GlcNAc and *N*, *N′*-diacetylchitobiose, with low degree of polymerization, has a better effect on promoting plant germination growth than many other reports, which is of great significance for increasing crop yields and increasing income.

## Conclusion

Here, the *ChiA* gene from *B. licheniformis* has been highly expressed in *P. pastoris*. The activity of ChiA in the fermentation supernatant reached 168.78 U/mL with 12.7 mg/mL protein, which is the highest data reported until now. The recombinant ChiA can hydrolyze 30% collodidal chitin into GlcNAc and *N*, *N*′-diacetylchitobiose with high conversion ratio. Combined with BsNagZ, GlcNAc can be obtained as the only product. Additionally, the hydrolysate of ChiA alone or combining with BsNagZ can obviously accelerate the germination growth of rice and wheat.

## Supplementary information

**Additional file 1: Table S1.** The effects of different hydrolyzate on the germination of rice and wheat seeds. **Fig. S1.** Construction of pHBM905M-*ChiA* recombinant plasmids. AOX1: d1 + 2 × 201 AOX1 promoter; α-factor: MF4I-SS signal peptide; TT: AOX1 terminator. **Fig. S2.** Construction of multi-copy the target gene expression cassettes based on the pHBM905BDM vector. (A) Schematic diagram of constructing recombinant plasmids containing two copy *ChiA* expression cassettes. (B) *Sal*I digestion of multi-copy expression plasmids. lane M: λ-*Eco*T14 digestion marker; lane 1-5: The plasmids pHBM905M-*ChiA*-1copy, -2copy, -3copy, -4copy, and -6copy by *Sal*I digestion, respectively. **Fig. S3.** Construction of pGAPZB-*HAC1/ERV29/SEC16/COG5/TRM1* recombinant plasmids. PGAP: GAP promoter; AOX1 TT: AOX1 terminator; PTEF1: TEF1 promoter; PEM7: EM7 promoter; CYC1TT: CYC1 terminator. **Fig. S4.** Relationship between copy number and enzyme activity. The data used is the highest enzyme activity in per copy number.

## Data Availability

The data collected upon which this article is based upon are all included in this manuscript.
