# Supplementary material for: Multiple strategies to improve the yield of chitinase a from Bacillus licheniformis in Pichia pastoris to obtain plant growth enhancer and GlcNAc
Source: Microb Cell Fact. 2020 Sep 15;19:181. doi: 10.1186/s12934-020-01440-y (PMC7493387; doi:10.1186/s12934-020-01440-y)
Supplement: Supplementary file 1 — Additional file 1: Table S1. The effects of different hydrolyzate on the germination of rice and wheat seeds. Fig. S1. Construction of pHBM905M-ChiA recombinant plasmids. AOX1: d1 + 2 × 201 AOX1 promoter; α-factor: MF4I-SS signal peptide; TT: AOX1 terminator. Fig. S2. Construction of multi-copy the target gene expression cassettes based on the pHBM905BDM vector. (A) Schematic diagram of constructing recombinant plasmids containing two copy ChiA expression cassettes. (B) SalI digestion of multi-copy expression plasmids. lane M: λ-EcoT14 digestion marker; lane 1-5: The plasmids pHBM905M-ChiA-1copy, -2copy, -3copy, -4copy, and -6copy by SalI digestion, respectively. Fig. S3. Construction of pGAPZB-HAC1/ERV29/SEC16/COG5/TRM1 recombinant plasmids. PGAP: GAP promoter; AOX1 TT: AOX1 terminator; PTEF1: TEF1 promoter; PEM7: EM7 promoter; CYC1TT: CYC1 terminator. Fig. S4. Relationship between copy number and enzyme activity. The data used is the highest enzyme activity in per copy number. [file 12934_2020_1440_MOESM1_ESM.docx]

# Additional file 1:

# Table. S1 The effects of different hydrolyzate on the germination of rice and wheat seeds

| Plant | Growth length (cm) | H_2_O | Phosphate  buffer | Increasing percent (%) | Hydrolysisproducts by ChiA | Increasing percent (%) | Hydrolysis product by ChiA /BsNagZ | Increasing percent (%) |
| --- | --- | --- | --- | --- | --- | --- | --- | --- |
| Rice | Seeding | 3.8±0.4 | 4±0.3 | 5 | 6.5±0.7** | 71 | 5.5±0.6* | 45 |
|  | Root | 3.2±1.2 | 3.6±0.5 | 12 | 7±1.1* | 119 | 6±1.0 | 87 |
| Wheat | Seeding | 3.5±1.3 | 3.7±0.2 | 5 | 6.4±1.5 | 83 | 4.6±0.3 | 31 |
|  | Root | 3.2±0.9 | 3.1±0.7 | -3 | 5.1±0.9 | 59 | 4.8±0.8 | 50 |

300 μg/ml hydrolysate for rice and 10 μg/ml for wheat.

*P＜0.05(significant difference)，**P＜0.01(Extremely significant difference)

**Figure legends**

**Fig. S1** Construction of pHBM905M-*ChiA* recombinant plasmids. AOX1: d1 + 2×201 AOX1 promoter; α-factor: MF4I-SS signal peptide; TT: AOX1 terminator.

**Fig. S2** Construction of multi-copy the target gene expression cassettes based on the pHBM905BDM vector. (A) Schematic diagram of constructing recombinant plasmids containing two copy *ChiA* expression cassettes. (B) *Sal*I digestion of multi-copy expression plasmids. lane M: λ-*Eco*T14 digestion marker; lane 1-5: The plasmids pHBM905M-*ChiA*-1copy, -2copy, -3copy, -4copy, and -6copy by *Sal*I digestion, respectively.

**Fig. S3** Construction of pGAPZB*-HAC1/ERV29/SEC16/COG5/TRM1* recombinant plasmids. PGAP: GAP promoter; AOX1 TT: AOX1 terminator; PTEF1: TEF1 promoter; PEM7: EM7 promoter; CYC1TT: CYC1 terminator.

**Fig. S4** Relationship between copy number and enzyme activity. The data used is the highest enzyme activity in per copy number.

**Additional file 1: Figure**

**Fig. S1**


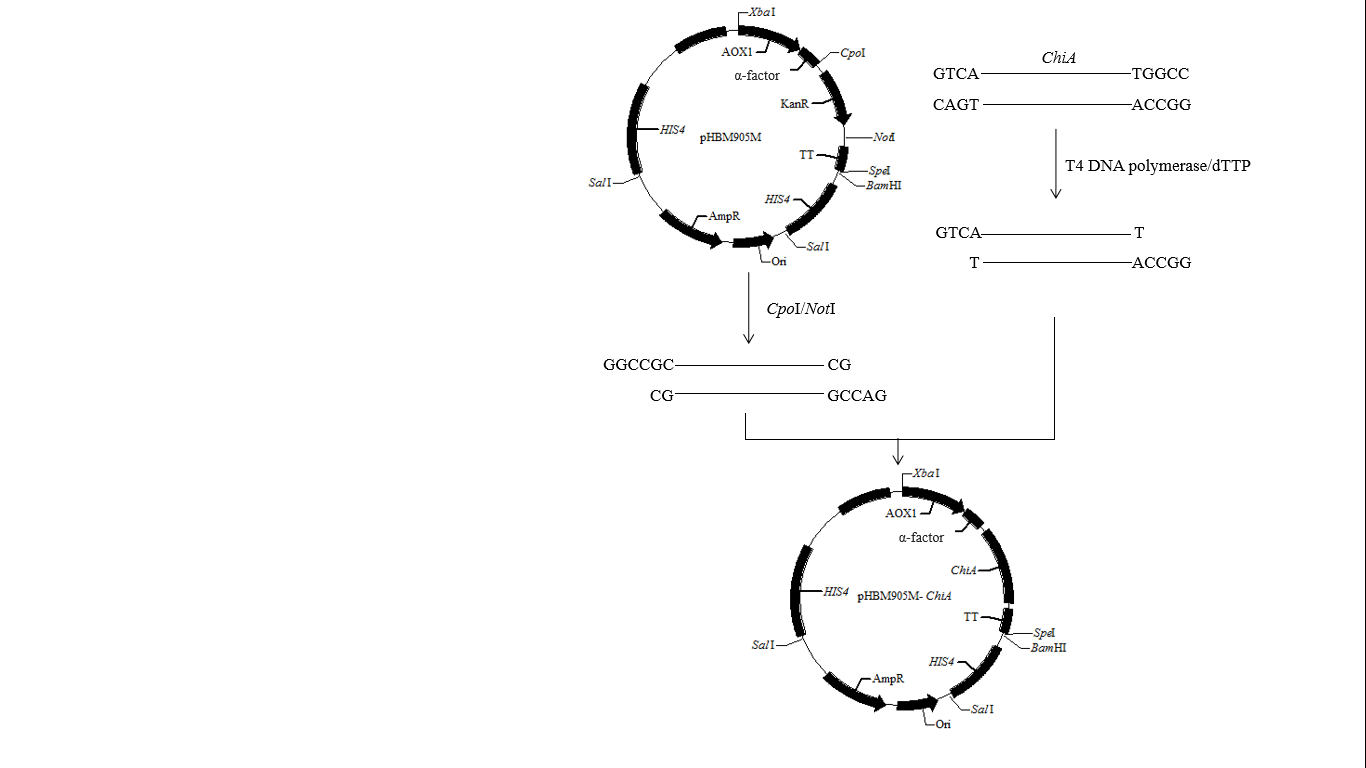


**Fig. S2**


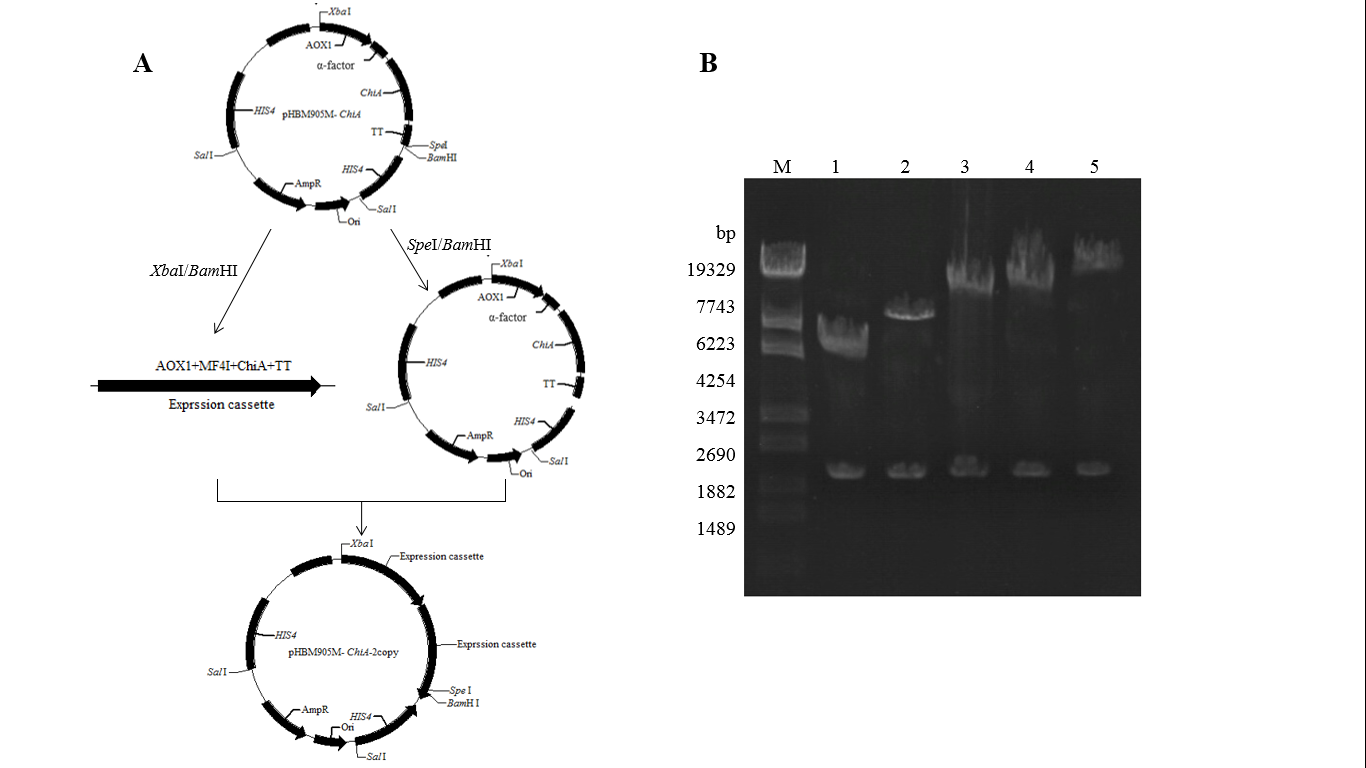


**Fig. S3**


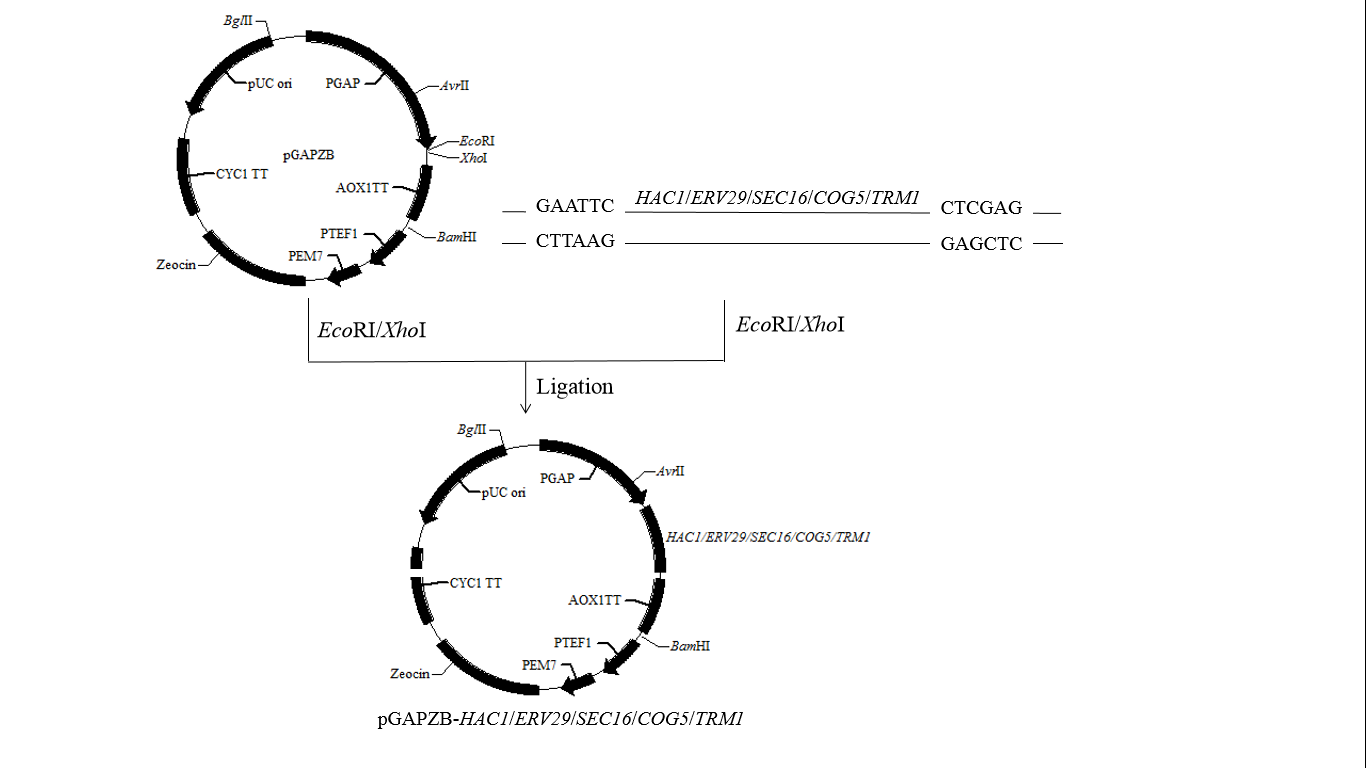


**Fig. S4**

**
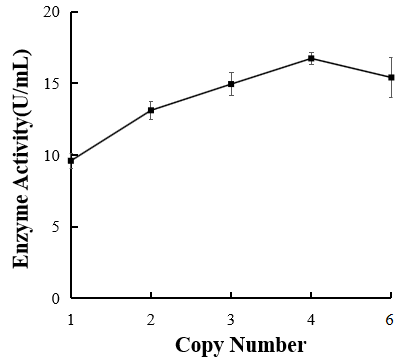
**
